# GOntoSim: a semantic similarity measure based on LCA and common descendants

**DOI:** 10.1038/s41598-022-07624-3

**Published:** 2022-03-09

**Authors:** Amna Binte Kamran, Hammad Naveed

**Affiliations:** Computational Biology Research Lab, Department of Computer Science, National University of Computer & Emerging Sciences (NUCES-FAST), Islamabad, 44800 Pakistan

**Keywords:** Gene ontology, Computational biology and bioinformatics, Functional clustering

## Abstract

The Gene Ontology (GO) is a controlled vocabulary that captures the semantics or context of an entity based on its functional role. Biomedical entities are frequently compared to each other to find similarities to help in data annotation and knowledge transfer. In this study, we propose GOntoSim, a novel method to determine the functional similarity between genes. GOntoSim quantifies the similarity between pairs of GO terms, by taking the graph structure and the information content of nodes into consideration. Our measure quantifies the similarity between the ancestors of the GO terms accurately. It also takes into account the common children of the GO terms. GOntoSim is evaluated using the entire Enzyme Dataset containing 10,890 proteins and 97,544 GO annotations. The enzymes are clustered and compared with the Gold Standard EC numbers. At level 1 of the EC Numbers for Molecular Function, GOntoSim achieves a purity score of 0.75 as compared to 0.47 and 0.51 GOGO and Wang. GOntoSim can handle the noisy IEA annotations. We achieve a purity score of 0.94 in contrast to 0.48 for both GOGO and Wang at level 1 of the EC Numbers with IEA annotations. GOntoSim can be freely accessed at (http://www.cbrlab.org/GOntoSim.html).

## Introduction

**Figure 1:**
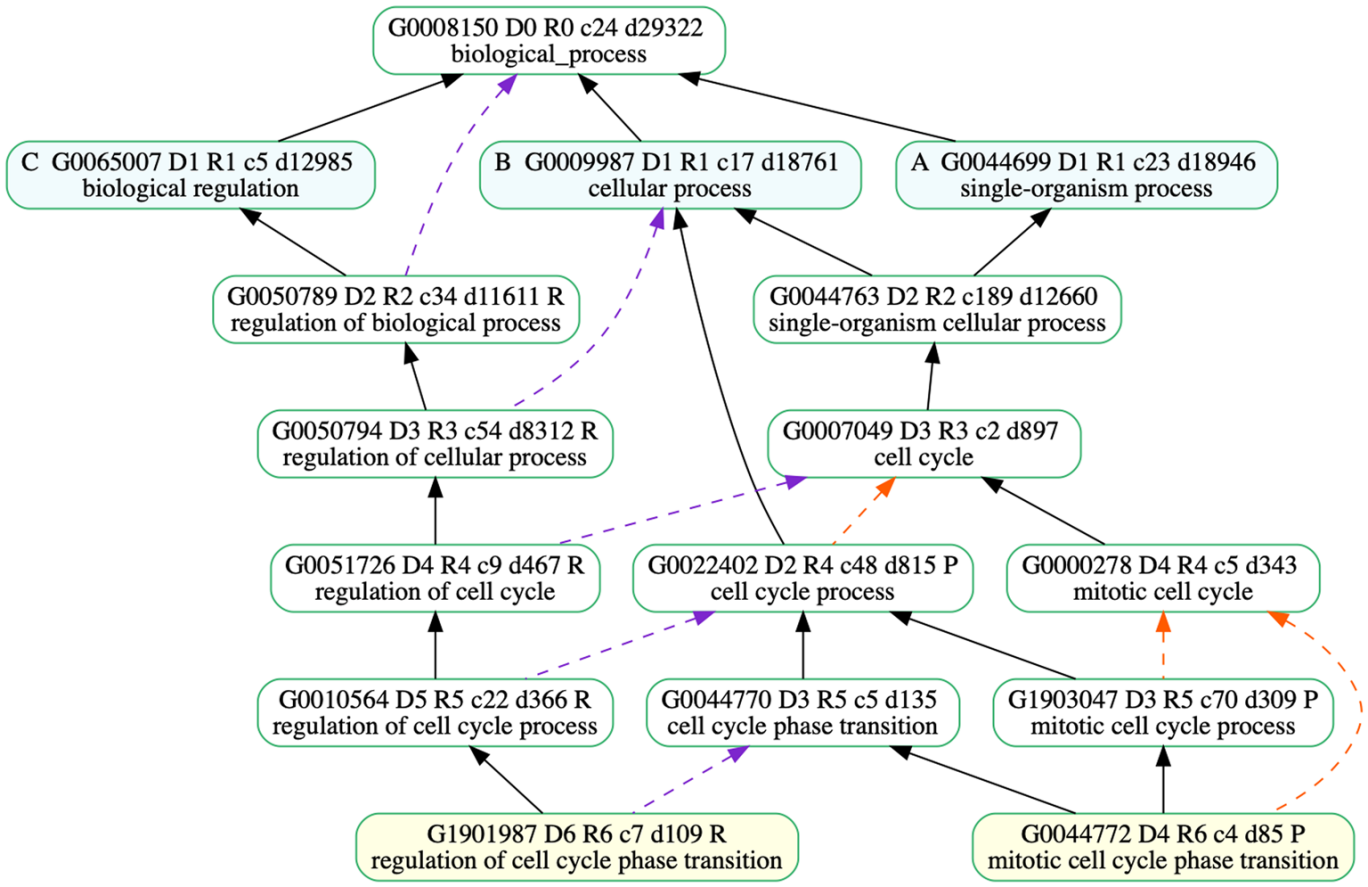
Part of GO graph of the BP category for the term “GO:1901987 (regulation of cell cycle phase transition)”- illustrating different relationships in GO. Figure drawn using goatools^4^ - version: 1.1.6 , https://github.com/tanghaibao/goatools, last accessed November 25th, 2021.

Biomedical entities, such as proteins or chemical compounds, are frequently compared to each other to find similarities^1^. Similarities can result in automated data annotation and knowledge transfer. In many cases entities with similar structures or sequences tend to behave in similar ways or have similar roles. However, there are many exceptions. A common example is that of caffeine and adenosine, which have similar shapes and caffeine can bind to adenosine receptors^2^. However, adenosine induces sleep and suppresses arousal while caffeine makes you more awake and less tired^2^. Semantic similarity is a way to measure the similarity of entities based on their function or context rather than their shape or physical characteristics. Over the past few decades, there has been an increasing use of ontologies to capture the semantics of entities. This is because the structure and information contained in ontologies and their annotations make them valuable for knowledge extraction. The Gene Ontology project provides a controlled vocabulary to describe gene and gene product attributes in any organism^3^. Gene Ontology is a schema for representing gene product function in the cellular context. It is structured as three independent Directed Acyclic Graphs (DAG) that correspond to orthogonal categories of gene product function: Molecular Function (MF), Biological Process (BP), and Cellular Component (CC). The nodes in the graph represent terms that describe components of gene product function. The GO terms are linked by relationships is_a, part_of, regulates (and +/− regulates), occurs_in, and has_part. Gene products that are described by GO terms are said to be annotated with them, either directly or through inheritance, since annotation to a given term implies annotation to all of its ancestors (true path rule). Figure 1 shows a part of the GO graph of the BP category for the term “GO:1901987 (regulation of cell cycle phase transition)”, where “GO:1901987” is its term ID and “regulation of cell cycle phase transition” is its descriptive axiom. The term “GO:1901987” can be traced to the root term “GO:0008150 (biological process).” The black, red, and purple arrows are indicative of relationships is_a, part_of, and regulates respectively.

The problem of measuring semantic similarity has been extensively studied in the literature. Several semantic similarity measures have been proposed for the comparison of terms in a graph-structured ontology. These measures can be divided broadly into node-based or edge-based methods. The node-based techniques compare the properties of the nodes of the ontology, usually with the help of the Information Content (IC) of a term. The IC of a term T is computed from a large corpus as the negative log of the likelihood of probability,

1 $$\begin{aligned} IC\ \left( T\right) =\ -\log {(P\left( T\right) )} \end{aligned}$$

where P(T) is the probability of occurrence of T in a specific corpus (such as the UniProt Knowledge-base), usually estimated by its frequency of annotation. Several IC-based methods have been applied to the Gene Ontology, most common of which (Resnik’s, Lin’s, and Jiang and Conrath’s) were initially proposed for Natural Language Processing using the WordNet ontology^5–7^. Resnik calculates the similarity of two terms by computing the IC of their most informative common ancestor (MICA). Lin adds to this measure by including the IC of the terms themselves with that of their MICA. Jiang and Conrath calculate similarity by incorporating the distance between the terms and IC of their MICA where the distance between the nodes is the shortest path linking the two nodes/terms. The measures by Resnik and Lin consider only is_a relationships. Lord et al. present a preliminary study where they show that Resnik works best for is-a hierarchies and state that these measures can be expanded to more complex ontologies by handling other types of relationships^8^. It might be possible to extend the applicability of Resnik’s measure to other relationships by assigning different weights to these relationships. However, nobody has yet demonstrated this comprehensively.

On the other hand, the edge-based measures rely on the number of edges connecting the nodes. Different measures take different properties like shortest path, depth of the term, the common path from the terms to the root, or distance between the terms^9, 10^. Wu and Palmer introduce a new measure that takes into account the Lowest Common Ancestor (LCA) of the two terms and calculates the similarity between them using the depth of the terms,

2 $$\begin{aligned} {sim}_{A,\ B}= \frac{2*depth({LCA}_{A,B})}{depth\left( A\right) +depth(B)} \end{aligned}$$

Where the depth of a term is the shortest path from the term to the root node of the ontology^9^.

Over the years, several hybrid measures were also introduced which combined the edge-based and node-based techniques^11–14^. Wang calculates the structural IC of the GO terms using the different relationships of the edges connecting the terms to the root^11^. Nagar and Al-Mubaid compute the IC with the path length between two terms, the specificity of their most informative common ancestor (MICA) and a relative number of child terms in the ontology^12^. They apply a negative exponential to the path length between the two terms to capture the concept of increasing specificity deeper in the graph. Zhao assigns a weight to relationships in GO based on the type of relationship and the number of children of a GO term to calculate the semantic value or contribution of a GO term^14^.

More recently, several new measures were introduced which did not belong to either of these categories^13, 15–19^. Smaili proposes generating vector embedding for the Gene Ontology terms and calculating the cosine similarities between the vectors of the terms^16, 17^. Peng introduces a network-based approach that takes a GO term’s graph as a network and applies network-based techniques to find the similarities between different terms^18, 19^. The authors use a random walk with restart method to traverse the GO graph.

**Figure 2:**
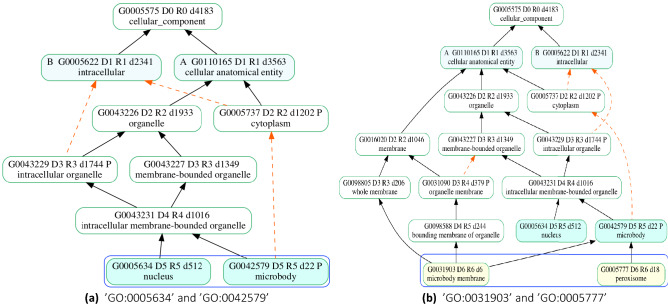
Graphs for comparison of GO Terms. In both cases, the two GO terms being compared are sibling nodes. (**a**) shows the terms with depth = 5 and (**b**) shows terms at depth = 6. Figures drawn using goatools^4^ - version: 1.1.6 , https://github.com/tanghaibao/goatools, last accessed November 25th, 2021.

Several limitations were observed in the existing semantic similarity measures. The node-based measures are often IC-based which leads to the similarities being affected by the annotation bias according to current research trends^20^. These measures are dependent on the statistics of an annotation corpus. These corpora keep changing leading to different similarity values for the same terms. The same GO terms being compared may have different similarity values if different gene annotation datasets are used. For the measures using the graph structure of the ontology, edge counting methods are common. These fail to capture the actual context of the terms because they either do not take into account the different relationships in the ontology or they assume that all edges have the same importance or that the terms at the same level have equal semantic distance^21^. Studies proposed to attenuate the above limitations often add the root node to the semantic contribution of a term and thus in the similarity between two terms. The direct child nodes of a root of an ontology are not similar, therefore should not be considered in the similarity calculations as done by Wang *et al.* and Zhao & Wang^11, 14^. Moreover, for Wang and GOGO, as we compare more specific terms that are deeper down the graph, the similarity scores decrease. The probable reason is that a deeper term means that there are many paths to the root which adds noise to the semantic contribution of the terms being compared. For example, when calculating the similarity between GO terms ’GO:0042579’ and ’GO:0005634’, Wang’s measure gives the similarity value of 0.74 but when we compare two terms deeper in the graph, ’GO:0031903’ and ’GO:0005777’, the similarity drops to 0.63. The DAGs for these terms are shown in Fig. 2. This contradicts the authors’ claim that their method produces a greater similarity value for the two terms deeper in the graph than that of the two terms closer to the root.

**Figure 3:**
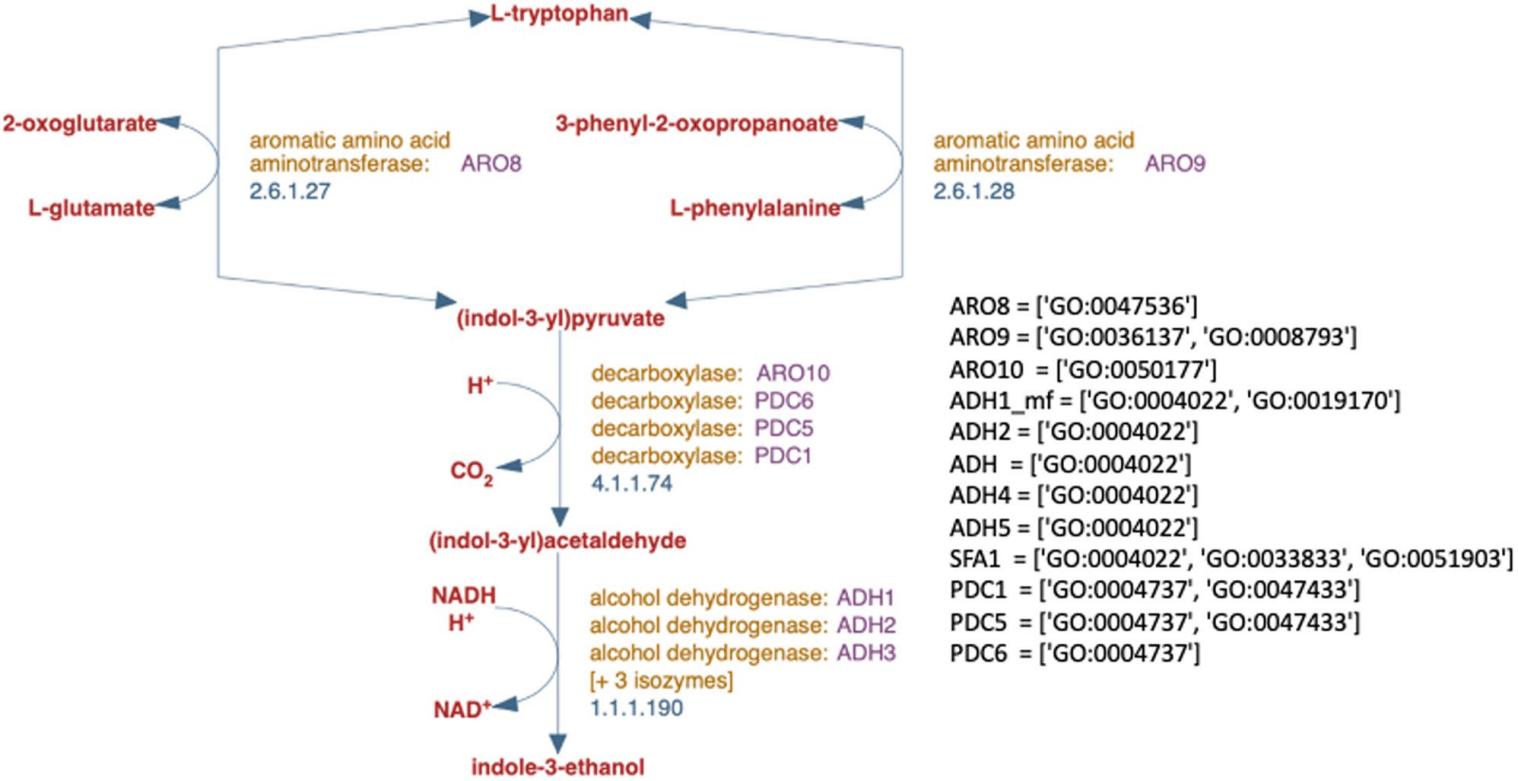
The tryptophan degradation pathway retrieved from the SGD database. There are 12 genes annotated with a total of 18 GO terms from Molecular Functions. For the EC number 1.1.1.190, 4 out of 5 genes are only annotated with a single MF GO term GO:0004022. This figure was made by modifying the image downloaded from the website of the SGD database.

The measure GOGO takes into account the number of children of a GO term when calculating similarity^14^. This does not seem to capture the true similarity because the number of child nodes does not indicate common children between the terms being compared. Moreover, the existing literature lacks a proper evaluation system for these similarity measures. Many studies show that their proposed method outperforms others by clustering gene pairs according to their measure and comparing them to the human perception^11, 14^. They chose the Saccharomyces Genome Database (SGD), which being manually curated provided the Ground Truth lacking in many other evaluation techniques. The genes in the SGD pathways have too few GO term annotations therefore, using them failed to provide an accurate picture of the similarity. The example in Fig. 3 shows that four out of six genes for EC Number 1.1.1.190 are all only annotated with a single GO term, ’GO:0004022’. They are being clustered together because they are annotated with the same GO term rather than because of the similarity measure. Other measures use Protein-Protein Interaction predictions or Gene Co-expression for evaluation of the semantic similarity measures^12, 16, 19^. However, the PPI predictions and the gene co-expression data both consist of predictive values that may not have enough credibility to be used as ground truths for an appropriate evaluation of similarities. Many measures in literature have been evaluated using a collaborative evaluation tool by Pesquita *et al.*^22^. A newer version was introduced as a collection of benchmark datasets^23^. However, they merely use proxy measures for protein similarity such as sequence similarity and protein-protein interactions (PPI) that are compared with the new semantic measures. The sequence similarity can not be used as an evaluation metric for semantic similarity because the association between them is nonlinear^24^. The PPI datasets indicate that if two proteins interact, they are similar because they will have common locations^23^. However, this does not imply their functional similarity which is being measured by the semantic similarity measures^23^.

In this study, we propose GOntoSim, a new hybrid measure to calculate the similarity between biological entities. The similarity between two Gene Ontology terms is calculated by encoding the context of these terms in a numeric value and taking into account the path with the maximum semantic value from their Lowest Common Ancestor (LCA) to the root of the ontology. We also incorporate the common children of the GO terms since they imply that the terms have further characteristics in common deeper in the graph which other measures fail to consider. We evaluate our measure on the first level of the Enzyme Commission Number (EC) from the Enzyme Dataset. We calculate the similarity between the enzymes using our measure and cluster them. We then compare the clusters according to the number of EC classes. We also compare our results with existing measures of Wang and GOGO^11, 14^. At level 1 of the EC Numbers for MF annotations, GOntoSim achieves a purity score of 0.75 ± 0.07 as compared to 0.51 ± 0.07 and 0.47 ± 0.05 for Wang and GOGO. This method also takes advantage of Inferred annotations (IEA) by achieving a purity score of 0.94 ± 0.07 in contrast to 0.48 ± 0.1 for both Wang and GOGO at level 1 of the EC Numbers with IEA annotations.

## Methods

**Figure 4:**
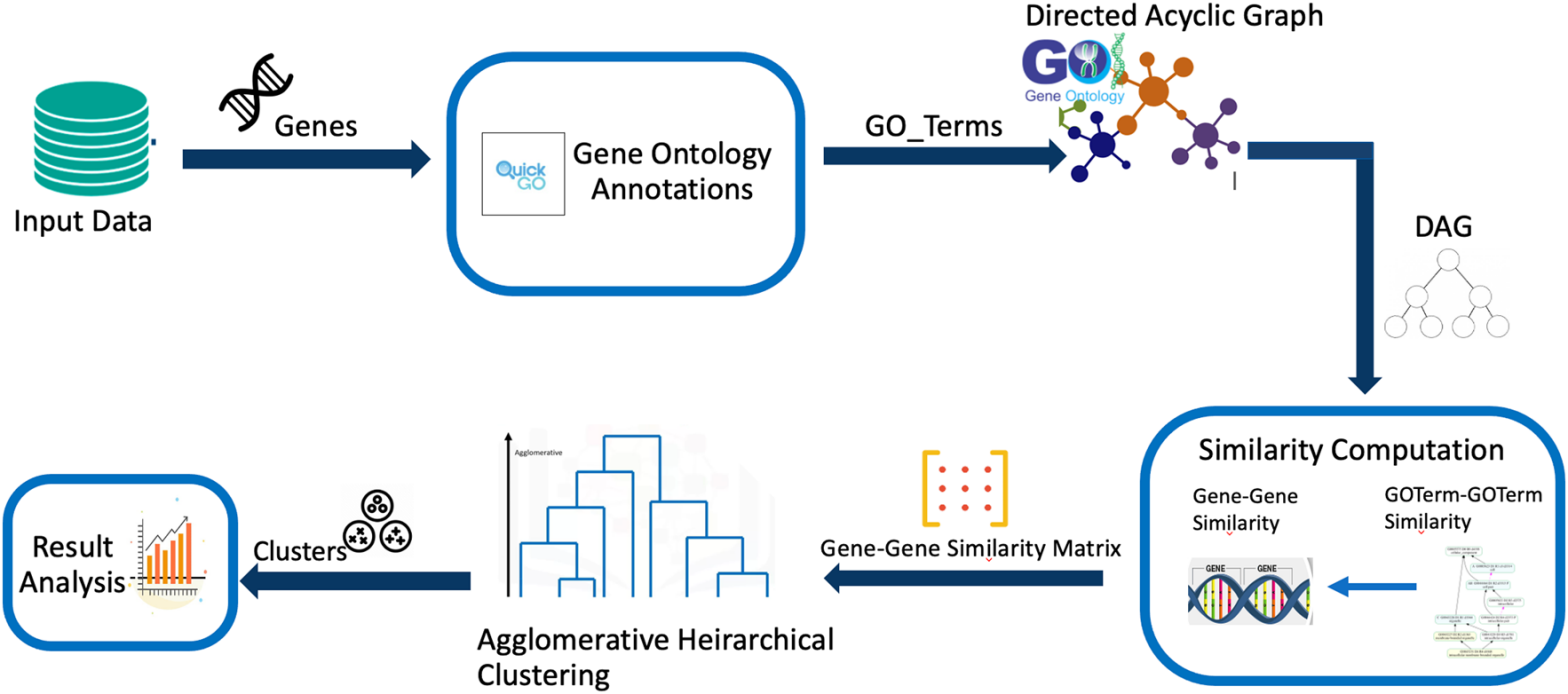
Methodology Framework. To find the similarity between genes, the GO annotations are downloaded using the Quick-GO API. A DAG is obtained for each GO term from the Gene Ontology.The similarity between genes is calculated and the resulting Gene-Gene similarity matrix is used to cluster the genes. The clusters are then evaluated for quality.

The methodology adopted for the research presented in this paper is shown in Fig. 4. To calculate the similarity between genes or biological entities, we first find the GO annotations for each entity using the Quick-GO API. Each entity is annotated with one or more GO term. Then for each term, a DAG is obtained from the Gene Ontology, which is used to calculate the similarity between the terms according to our similarity measure. Using the similarity between the terms and the Best Match Average (BMA) approach, we calculate the similarity between genes. The Gene-Gene similarity matrix is used to cluster the genes in hierarchical agglomerative clustering. The resulting clusters are then evaluated for quality.

### Baseline

The methods of Wang and GOGO produce the results that are closest to human perception of similarity among the existing methods^11, 14^. However, they both count the root of the ontology into the semantic values of the terms. We design a new baseline measure that computes semantic contributions of the term and its ancestors without counting the root. The semantic contribution of a term from its upward DAG is represented as $DAG_{A} = (A, T_{A}, E_{A})$ where $T_{A}$ is the set of GO terms for a given GO Term A and its ancestors, and $E_{A}$ is the set of edges linking the nodes of $T_{A}$ in $DAG_{A}$. Each edge in $E_{A}$ is assigned a weight ($W_{e}$) according to the type of relationship the edge represents in the graph. Here, we empirically set $W_{e}$ to be 0.8, 0.6, and 0.7 for the is_a, part_of, and regulates relationships respectively. In the semantic contribution of $DAG_{A}$, the GO term A is taken as 1, representing the maximum contribution. For all the rest of the terms in the DAG, the contribution of an ancestor term $t^{\prime }$ of A is a maximized product of all the edges in the path from A to $t^{\prime }$. The root of the ontology is considered to have zero semantic contribution for the term A.

3 $$\begin{aligned} S_{A}(t) = \left\{ \begin{array}{c} S_{A}\left( root\right) =0\\ S_{A}\left( A\right) = 1\\ \max \left\{ W_{e}\times S_{A} \left( t^{\prime }\right) | t^{\prime } \in children\ of(t)\right\} , \ if\ t\ne A \end{array}\right. \end{aligned}$$

The total semantic value SV(A) of a GO term A is the sum of all the semantic contributions of the term and its ancestors.

4 $$\begin{aligned} SV\left( A\right) =\ \sum _{t\ \in \ T_{A}}{S_{A}(t)} \end{aligned}$$

The Semantic Similarity between two GO terms is calculated by taking the sum of semantic contributions of intersecting terms divided by the sum of the total Semantic Values of the two terms.

5 $$\begin{aligned} Sim_{Baseline}\left( A,B\right) =\ \frac{\sum _{t\in T_{A} \cap T_{B}}{(S_{A}\left( t\right) +\ S_{B}(t))}}{SV\left( A\right) +SV(B)} \end{aligned}$$

### Baseline + descendants

To best utilize the graph structure of the Gene Ontology, we incorporate our baseline method into the contributions of common descendant terms. This similarity is calculated as:

6 $$\begin{aligned} {Sim}_{Baseline+Desc}\left( A,B\right) = \left( (Sim_{Baseline} \left( A,B\right) *U \right) + \left( Sim_{Desc} \left( A,B\right) *L\right) \end{aligned}$$

Where $Sim_{Baseline}$ is defined in Eq. (5) and $Sim_{Desc}$ in Eq. (9). U and L represent upward and downward similarities are to normalise the similarity value between 0 and 1. We tried two variations; One where the values of U and L are both 0.5 and the other where $\hbox {U}=0.7$ and $\hbox {L}=0.3$.

### GOntoSim

Analysis of our Baseline measure reveals that as we go down the Gene Ontology graph, the number of paths to the root increases. This adds noise to the final similarity of the two terms. GOntoSim calculates the similarity between two GO terms by using their lowest common ancestor (LCA) and common descendants. The method finds the LCA and the highest common descendant (HCD) of the given terms and then encodes the semantic contributions of the two GO Terms, their LCA, and their common descendants in a numerical value. The similarity is then computed in two stages. Eq.(3) is used to calculate the semantic contributions of the terms between a GO term A and the root. Then, the total semantic value of A is calculated as the sum of all the semantic contributions of the term and its ancestors on the path with the maximum sum. The semantic value of A is defined as:

7 $$\begin{aligned} SV\left( A\right) =\ max_{p\ \in \ P_{A}} \left( \sum _{t\ \in \ p}S_{A}(t)\right) \end{aligned}$$

where $P_{A}$ is the set of all paths from the GO term A to the root. The upward similarity between two GO terms is twice the Semantic value of the LCA of the two terms A and B, divided by the sum of the semantic values of A and B.

8 $$\begin{aligned} Sim_{LCA} \left( A,B\right) = \frac{2 * SV({LCA}_{A,B})}{{SV \left( A\right) + SV\left( B\right) }} \end{aligned}$$

The calculation of the semantic value of a GO term with its LCA (Eq. (8)) is influenced by the similarity measure by Wu and Palmer but modified to account for the different types of relationships between a term and its ancestors^9^. For the semantic contribution of a term with its lower DAG, we take the highest common descendant of the A and B and then measure the semantic contributions of the terms and their descendants. The downward similarity is measured using the contributions of the common children divided by the sum of all children of the two terms up to a specified depth, which is obtained by $depth_{HCD}$ - $depth_{A}$. Note that the depth of a node is the longest distance from the root. $A_{d}$ and $t_{d}$ represent the downward semantic contribution of the term A and a descendant node respectively.

9 $$\begin{aligned} Sim_{Desc}\left( A,B\right) =\frac{\sum _{t\in T_A\cap T_B} {(S_A\left( t_{d}\right) +\ S_B\left( t_{d}\right) )}}{SV\left( A_{d}\right) +SV(B_{d})} \end{aligned}$$

As shown in Eq. (10) the total similarity $({Sim}_{GOntoSim})$ between Terms A and B is the sum of the upper and lower similarities. In case there is no common descendant to the two terms, the downward similarity is assumed to be zero. To keep the similarity value between 0 and 1, we multiply the upper similarity and downward similarity with 0.5 each.

10 $$\begin{aligned} {Sim}_{GOntoSim}\left( A,B\right) = \left( (Sim_{LCA} \left( A,B\right) *0.5 \right) + \left( Sim_{Desc} \left( A,B\right) *0.5\right) \end{aligned}$$

### Baseline_LCA

To check the impact of the common descendants on GOntoSim, we implement another measure by removing the downward similarity from GOntoSim. This Baseline_LCA method calculates the similarity between two GO terms with the path from their lowest common ancestor (LCA) to the root only. This method shares the same implementation of our final method to find the semantic contribution of a $DAG_{A}$. For all paths from A to the root in $DAG_{A}$, the sum of all the semantic contributions of the term and its ancestors is calculated in Eq. (3). The path with the maximum sum is selected Eq. (7). The total semantic value or contribution of a GO term is the sum of all the semantic contributions of the term and its ancestors on the selected path. The Semantic Similarity between two GO terms as shown in Eq. (8) is the Semantic value of the LCA of the two terms divided by the average of their semantic values. This ensures that $0< Sim_{GO} (A,B) < 1$ always holds true.

### Similarity between genes

We use the Best Match Average strategy to measure the similarity between two Biological Entities. Each entity is considered as a group of GO terms. The similarity between the groups/sets is calculated by measuring the similarity of each GO term in one set with every GO term in the other set; this similarity is defined as the maximum semantic similarity between a Term from one set and any of the terms in the other set.

11 $$\begin{aligned} Sim\left( go,\ GO\right) =\ {max}_{1\le i\le k}(S_{GO}(go,{go}_i)) \end{aligned}$$

Functional Similarity between two genes or biological entities is the sum of semantic similarity between each term in one set with the other set and vice versa, divided by the total number of terms in the 2 sets.

12 $$\begin{aligned} Sim\left( G1,\ G2\right) =\ \frac{\sum _{1\le i\le m} {Sim({go}_{1i},{GO}_2)}+\ \sum _{1\le j\le n}{Sim({go}_{2j},{GO}_1)}}{m+n} \end{aligned}$$

### Dataset

To validate our methodology, we use all the entries for the six major enzyme classes from the Swiss-Prot database (downloaded in January 2019). Following Memon *et al.*^25^, the downloaded dataset was then cleaned by using the CD-HIT^26^ to obtain the sequences with a similarity threshold of 40% to ensure that the data was non-redundant. All the enzymes belonging to multiple classes were removed and only the sequences with the 20 naturally occurring amino acids were selected. Furthermore, all protein sequences with a length less than 50 and those with 1000 or more amino acids were removed. For each of these enzymes in our dataset, we used the Quick GO API to download all GO Term annotations (downloaded in January 2020). We filter these annotations according to their aspects (MF, BP, and CC) into separate datasets. These were further filtered for the evidence code IEA (Inferred from Electronic Annotation) because they have not been reviewed by experts. Datasets created by removing the IEA annotations are labeled NONIEA and datasets including them are labeled IEA. The Enzyme Dataset is manually curated and can be considered as the gold standard. True Labels are thus taken from the dataset itself. For the clustering on Level 1, we filter the enzymes in six classes and assign the respective first digit of the EC number as the true label for each enzyme.

For the evaluation of our similarity measure, we construct nine subsets from the original Enzyme dataset. Statistics of these subsets are shown in Table 1. There were a total of 55,274 MF GO term annotations for the Enzymes Dataset including ones with IEA evidence code. There were 7,279 MF annotations in the NONIEA subset. This is in contrast to the 18 annotations for the SGD pathway for Tryptophan Degradation.

**Table 1:** Number of Enzymes and their annotated GO terms for Level 1 of EC numbers.

	IEA		NONIEA	
	Enzymes	GO Terms	Enzymes	GO Terms
Level 1 - (MF)	10,890	55952	3416	7279
Level 1 - (BP)	10,614	41592	3116	10915

### Implementation

We downloaded the Gene Ontology - go-basic.obo file (rel(2020-01-01) - from the GO Consortium Website (http://www.geneontology.org/ontology/) in January, 2020. We use the GOATOOLS^4^ to read the Gene Ontology and parse it as a DAG; implementing GOntoSim by getting a DAG of each term and traversing all paths to the root to calculate the Semantic Value of a term. We use Python to code GOntoSim and the Scikit Learn library to implement the evaluation methodology for it.

We evaluate our similarity measures on different enzyme datasets described above and perform unsupervised agglomerative clustering based on the resulting similarities between them. We then validate the clusters on the evaluation metrics. The number of enzymes for each class varied greatly so we perform random sampling on each dataset for each cluster to have equal representation. We randomly take the same number of samples from each class, calculate their similarities, cluster them, and perform cluster validation. We repeat this process ten times and take the mean values of the cluster evaluation scores as well as their standard deviations to ensure quality in results being reported. The Ground truth to analyze the clustering results is taken from the corresponding levels of EC number assigned to each enzyme.

### Evaluation metrics

The clusters generated by the agglomerative hierarchical clustering are evaluated with several existing extrinsic measures for cluster evaluation. The measures that we use are Purity score, Adjusted Rand index, Fowlkes-Mallows scores, Mutual information-based scores, Homogeneity, Completeness, and V-measure scores. All of these measures require ground truth labels which are the true labels generated from the dataset. Agglomerative Clustering performs a hierarchical clustering using a bottom-up approach: each observation starts in its cluster, and clusters are successively merged. The linkage criteria determines the metric used for the merge strategy. We use the maximum or complete linkage which minimizes the maximum distance between observations of pairs of clusters. Further details about these evaluation metrics are provided in the supplementary SI-2.

## Results and discussion

In this section, we present the results of our experiments carried out on the Enzyme Datasets. To compare our results we analyzed GOntoSim, our baseline measure, baseline$+$descendants, baseline_lca as well as the measures by Resnik, Lin, Wang, and GOGO across different subsets of the Enzyme dataset like IEA and non-IEA, MF and BP^5, 6, 11, 14^.

### Performance on MF-NonIEA enzymes

**Figure 5:**
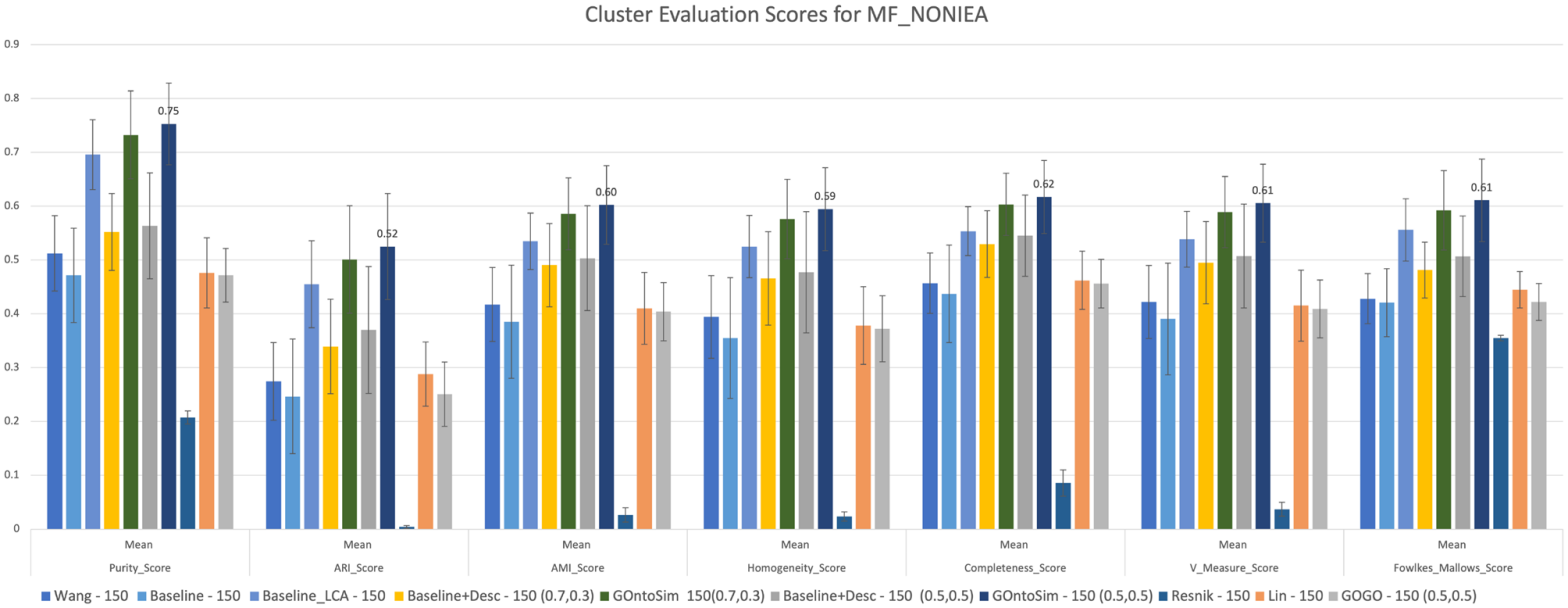
Cluster Evaluation at EC Level 1 for MF annotations (NONIEA). GOntoSim Method with equal upper and lower weights to semantic contributions achieves the highest purity score of 0.75 ± 0.07.

In our first experiment, we took all the enzymes, downloaded their MF annotations, filtered out the annotations with the evidence code ’IEA’. We found the similarity between all pairs of genes. We then clustered them into six clusters to simulate the six classes at EC Level 1. Figure 5 shows that the GOntoSim Method with equal upper and lower weights to semantic contributions has the highest purity score of 0.75 ± 0.07 whereas GOntoSim with different weights 0.7 and 0.3 for the upper and lower semantic contributions respectively has the second-highest purity score of 0.73 ± 0.06. All the other metrics namely Adjusted Rand index, Fowlkes-Mallows score, Mutual information-based score, Homogeneity, Completeness, and V-measure scores follow the same pattern where GOntoSim clearly outperforms the rest of the measures. In this experiment, we randomly sample 150 observations from each cluster. Similar trends are found when 50 or 100 samples are taken. (See Table 1 from Supplementary Information for details.)

### Performance on BP-NonIEA enzymes

**Figure 6:**
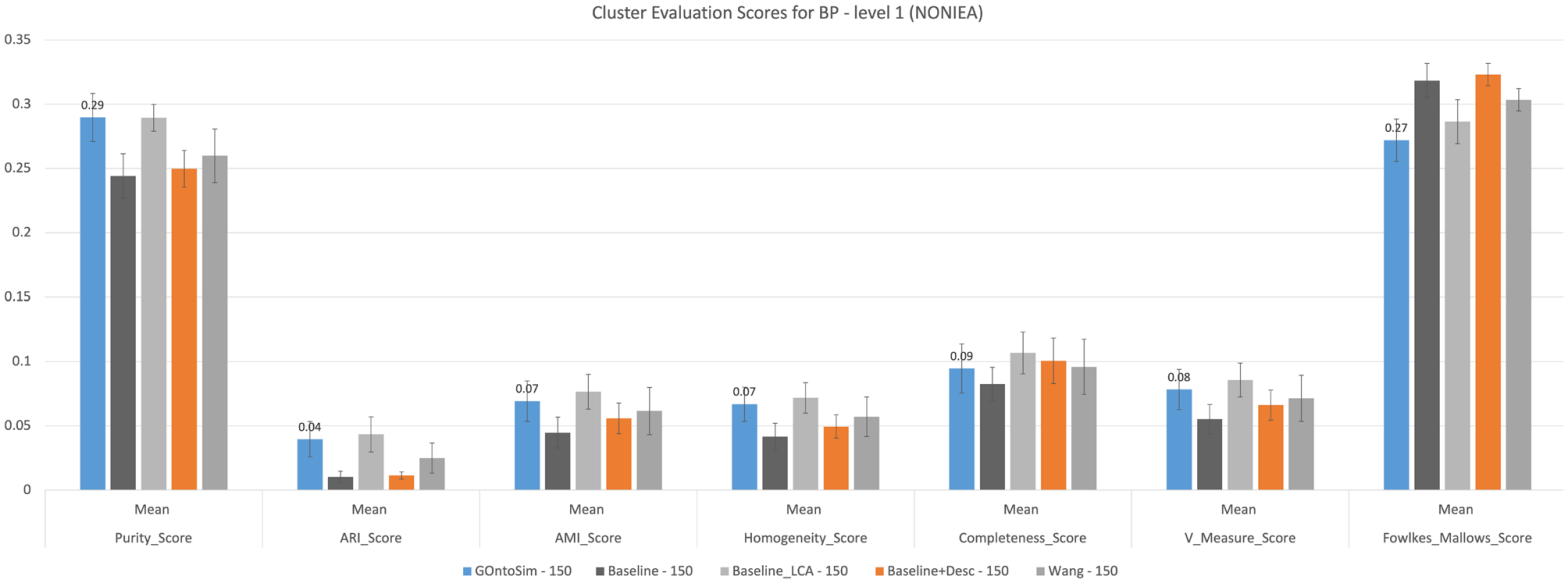
Cluster Evaluation at EC Level 1 for BP annotations (NONIEA). GOntoSim Method with equal upper and lower weights to semantic contributions achieves the highest purity score of 0.29 ± 0.02.

In our second experiment, we select a similar subset as the first experiment but select BP annotations instead of MF ones. The overall cluster quality of BP annotations has decreased dramatically across all measures and clustering metrics. Figure 6 shows GOntoSim has the highest purity score of 0.29 ± 0.02 with 150 samples from each class. Baseline_LCA performs best from among these across all other metrics. (See Table 2 from Supplementary Information.)

### Performance on MF-IEA enzymes

**Figure 7:**
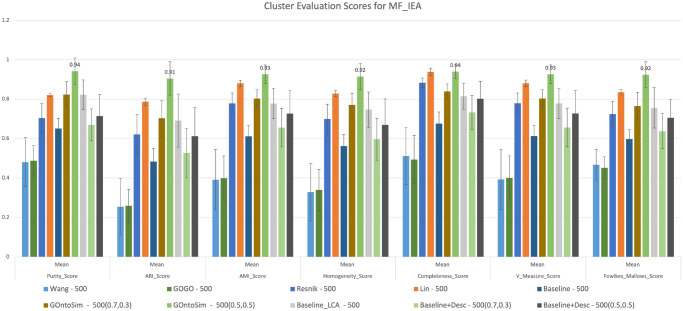
Cluster Evaluation at EC Level 1 for MF annotations (IEA). GOntoSim Method with equal upper and lower weights to semantic contributions achieves the highest purity score of 0.9 ± 0.06.

For our third experiment, we add the IEA annotations to the MF annotation subset from our first experiment, resulting in a much larger dataset. These inferred annotations are not manually reviewed, so they add some noise to the data. Most measures are unable to handle this noise. As shown in Fig. 7, at Level 1 for MF, Wang and GOGO both have decreased purity scores of 0.48 ± 0.1 for the IEA dataset as compared to 0.5 ± 0.07 for NONIEA. However, GOntoSim, with equal weights for upper and lower semantic contributions achieves a purity score of 0.94 ± 0.06. Different weights for upper and lower semantic contributions resulted in a purity score of 0.82 ± 0.06. These scores have increased from 0.75 ± 0.07 and 0.73 ± 0.06 for NONIEA. This trend remains consistent across all evaluation metrics. Increased purity scores and other metrics means that the IEA annotations increases the clustering quality. This is unique to GOntoSim as all other semantic similarity measures suffer a decrease in clustering quality after adding IEA annotations. (See Table 3 from Supplementary Information.)

### Automatic annotations

Ontologies, annotations, and classification schemes are consistently evolving. For example, class 7 of the EC numbers was introduced in August 2018. We wanted to test the usefulness of our measure in automatic annotation. Our test dataset only considers 6 classes of the EC numbers. We calculate the similarities between the 155 enzymes in class 7 at level 1 of the EC numbers. We use the GO term annotations of these enzymes downloaded from the Uniprot-GOA website before August 2018, when this new class was defined. At the time these enzymes belonged to class 3 at level 1. The average similarity between the enzymes of class 7 is 0.76 with a standard deviation of 0.1. On the other hand, the average similarity between 155 enzymes randomly selected from classes 1 to 6 is 0.56 with a standard deviation of 0.05. This shows that GOntoSim can be applicable for automatic fine-tuning of annotations.

### Optimizations

$Sim_{Baseline}$ in Eq. (5) and $Sim_{Desc}$ in Eq. (9), both have the term in the numerator, sum(SA(t)+SB(t)) included in both terms of the denominator, SV(A) and SV(B). this means that sum, (SA(t)+SB(t)) essentially appears twice in the denominator. In order to check whether the performance of our measure is not driven down by the similarity value for more similar pairs, we implemented our Baseline and GontoSim Measures with the Tanimoto Coefficient which uses the ratio of the intersecting set to the union set as the measure of similarity in Eq. (5) and in Eq. (9). We used the MF-IEA Enzymes data as well as the MF-NONIEA subset in the same manner as described above and found no significant change in the performance (details in SI).

### Future work

In the future, we aim to extend the application of our semantic similarity measure by considering other biomedical ontologies for similarity. In particular, we plan to apply GOntoSim to the Disease Ontology (DO)^27^. The DO is a knowledge base of human diseases connecting biological data with a disease-centered perspective. The similarity measures by Resnik, Lin and Wang have been used to calculate the similarity between the DO terms by Yu et al.^5, 6, 11, 28^. Finding the similarity among diseases could help researchers in obtaining information about human diseases for example by revealing common attributes which could improve the disease diagnoses^29^.

## Conclusion

GOntoSim is a semantic similarity measure that takes advantage of the graph structure of the Gene Ontology. This method finds the similarity between two GO terms with the context of their lowest common ancestor and their common descendants. With GOntoSim, we get the advantages of not depending on any external corpus, fully utilizing the common ancestor and common descendants of the GO terms being compared, while avoiding the noise added by too many paths to the root. We extensively evaluate our method using the entire Enzyme Dataset and compare our results with the Gold Standard EC numbers as well as two similar graph-based measures. Results show that GOntoSim clearly outperforms all other measures in the biological domain. At level 1 of the EC Numbers for Molecular Functions, we achieve a purity score of 0.75 ± 0.07 as compared to 0.51 ± 0.07 and 0.47 ± 0.05 for Wang and GOGO. This method also takes advantage of the IEA annotations as well as handles the noise added by them. We achieve a purity score of 0.94 ± 0.07 in contrast to 0.48 ± 0.1 for both Wang and GOGO at level 1 of the EC Numbers with IEA annotations. GOntoSim can be applied as an evaluation metric to assess the quality of automated annotations or to improve the annotations of entities based on the similarity of their biological functions.

## Supplementary Information

Supplementary Information.
