# Supplementary material for: GOntoSim: a semantic similarity measure based on LCA and common descendants
Source: Sci Rep. 2022 Mar 9;12:3818. doi: 10.1038/s41598-022-07624-3 (PMC8907294; doi:10.1038/s41598-022-07624-3)
Supplement: Supplementary file 1 — Supplementary Information. [file 41598_2022_7624_MOESM1_ESM.pdf]

Supplementary Information

Abbreviations

- GO: Gene Ontology;
- BP: Biological Process;
- CC: Cellular Component;
- MF: Molecular Function;
- DAG: Directed Acyclic Graph;
- EC: Enzyme Commission;
- IC: Information Content;
- LCA: Lowest Common Ancestor;
- MICA: Most Informative Common Ancestor;
- IEA: Inferred from Electronic Annotation;
- LFC: Logged Fold Change;
- BMA: Best Match Average;
- SSM: Semantic Similarity Measure;
- SGD: Saccharomyces Genome Database;
- PPI: Protein-Protein Interaction;
- RWR: Random Walk with Restart;
- SVD: Singular Value Decomposition;
- DO: Disease Ontology;

Evaluation Metrics

- Purity is a measure of the extent to which clusters contain a single class.

$$Purity = \frac{1}{N} \sum_{m \in D} max_{d \in D} |m \cap d| \tag{1}$$

- Adjusted Rand index Score measures the similarity of the two assignments, ignoring permutations and with chance normalization. If C is a ground truth class assignment and K the clustering, a the number of pairs of elements that are in the same set in C and in the same set in K b, the number of pairs of elements that are in different sets in C and in different sets in K

  The unadjusted Rand index is then given by:

$$\mathrm{RI} = \frac{a+b}{C_2^{n_{samples}}} \tag{2}$$

  where $C_2^{n_{samples}}$ is the total number of possible pairs in the dataset. It does not matter if the calculation is performed on ordered pairs or unordered pairs as long as the calculation is performed consistently.

  However, the Rand index does not guarantee that random label assignments will get a value close to zero (esp. if the number of clusters is in the same order of magnitude as the number of samples). To counter this effect we can discount the expected RI $E[RI]$ of random labelings by defining the adjusted Rand index as follows:

$$\mathrm{ARI} = \frac{\mathrm{RI} - E[\mathrm{RI}]}{\max(\mathrm{RI}) - E[\mathrm{RI}]} \tag{3}$$

- Fowlkes-Mallows score is the Geometric Mean between Precision and Recall.

$$\mathrm{FMI} = \frac{\mathrm{TP}}{\sqrt{(\mathrm{TP}+\mathrm{FP})(\mathrm{TP}+\mathrm{FN})}} \tag{4}$$

  Here,

  - True Positive: the number of pair of points belonging to the same clusters in true as well as predicted labels both.
  - False Positive: number of pair of points belonging to the same clusters in true labels but not in the predicted labels.
  - False Negative: number of pair of points belonging to the same clusters in the predicted labels but not in the true labels.

- Adjusted Mutual information-based score Measures the agreement of the two assignments, ignoring permutations, and is adjusted to account for chance.

$$\mathrm{MI}(U,V) = \sum_{i=1}^{|U|}\sum_{j=1}^{|V|} \frac{|U_i \cap V_j|}{N} \log\left(\frac{N|U_i \cap V_j|}{|U_i||V_j|}\right) \tag{5}$$

$$H(U) = -\sum_{i=1}^{|U|} P(i)\log(P(i)) \tag{6}$$

$$H(V) = -\sum_{j=1}^{|V|} P'(j)\log(P'(j)) \tag{7}$$

$$\mathrm{NMI}(U,V) = \frac{\mathrm{MI}(U,V)}{\mathrm{mean}(H(U),H(V))} \tag{8}$$

$$\mathrm{MI}(U,V) = \sum_{i=1}^{|U|}\sum_{j=1}^{|V|} P(i,j)\log\left(\frac{P(i,j)}{P(i)P'(j)}\right) \tag{9}$$

$$\text{E[MI(U,V)]=}\sum_{i=1}^{|U|}\sum_{j=1}^{|V|}\sum_{n_{ij}=(a_i+b_j-N)^+}^{\min(a_i,b_j)} \frac{n_{ij}}{N}\log\left(\frac{N.n_{ij}}{a_ib_j}\right)\frac{a_i!b_j!(N-a_i)!(N-b_j)!}{N!n_{ij}!(a_i-n_{ij})!(b_j-n_{ij}(N-a_i-b_j+n_{ij})!} \tag{10}$$

$$\mathrm{AMI} = \frac{\mathrm{MI}-E[\mathrm{MI}]}{\mathrm{mean}(H(U),H(V))-E[\mathrm{MI}]} \tag{11}$$

- Homogeneity, A clustering result satisfies homogeneity if Each cluster contains only members of a single class.

$$h = 1 - \frac{H(C|K)}{H(C)} \tag{12}$$

$$H(C|K) = -\sum_{c=1}^{|C|}\sum_{k=1}^{|K|} \frac{n_{c,k}}{n}\cdot\log\left(\frac{n_{c,k}}{n_k}\right) \tag{13}$$

$$H(C) = -\sum_{c=1}^{|C|} \frac{n_c}{n}\cdot\log\left(\frac{n_c}{n}\right) \tag{14}$$

- Completeness A clustering result satisfies Completeness if All members of a given class are assigned to the same cluster.

$$c = 1 - \frac{H(K|C)}{H(K)} \tag{15}$$

- V-measure Score is the Harmonic mean between homogeneity Score and Completeness Score. It can be used to evaluate the agreement of two independent assignments on the same dataset.

$$v = 2\cdot\frac{h\cdot c}{h+c} \tag{16}$$

Results

Table 1. NONIEA annotations for EC Level 1 - Molecular Function

MF(Level 1)	purity_score		ARI_Score		AMI_Score		homogeneity_score		completeness_score		v_measure_score		fowlkes_mallows_score	
samples * 10	Mean	Std-dev	Mean	Std-dev	Mean	Std-dev	Mean	Std-dev	Mean	Std-dev	Mean	Std-dev	Mean	Std-dev
Wang - 50	0.5737	0.0673	0.3413	0.072	0.4819	0.0724	0.4721	0.0748	0.5218	0.0684	0.4952	0.0704	0.4706	0.0528
Wang - 100	0.5302	0.072	0.3105	0.0762	0.4653	0.0737	0.4408	0.0813	0.5101	0.0597	0.4721	0.0725	0.4562	0.0474
Wang - 150	0.5122	0.0701	0.2743	0.072	0.4172	0.0685	0.394	0.077	0.4565	0.0562	0.422	0.0678	0.428	0.0465
GOGO - 50	0.532	0.0611	0.3063	0.0839	0.4585	0.0761	0.4444	0.0794	0.5066	0.0658	0.4729	0.0735	0.4539	0.057
GOGO - 100	0.467	0.0598	0.2334	0.0576	0.3866	0.0698	0.361	0.0737	0.4378	0.0569	0.3948	0.0684	0.4077	0.0279
GOGO - 150	0.4713	0.0499	0.2503	0.0597	0.4037	0.0543	0.372	0.0613	0.456	0.0453	0.4089	0.0537	0.4219	0.0343
Resnik - 50	0.4343	0.154	0.2465	0.1712	0.3732	0.2084	0.3334	0.1951	0.5011	0.1942	0.391	0.2001	0.4741	0.0802
Resnik - 100	0.2422	0.0537	0.0458	0.0795	0.0933	0.1226	0.0781	0.096	0.1885	0.1489	0.1073	0.1197	0.3739	0.033
Resnik - 150	0.2071	0.0121	0.0043	0.0026	0.0264	0.0132	0.0236	0.0086	0.0853	0.0243	0.0369	0.0129	0.3547	0.005
Lin - 50	0.5083	0.063	0.295	0.0659	0.4384	0.0749	0.4197	0.0748	0.4945	0.0684	0.4537	0.0723	0.4455	0.0443
Lin - 100	0.5203	0.059	0.3365	0.0912	0.4657	0.0784	0.4396	0.0849	0.5144	0.0627	0.4729	0.0768	0.4781	0.0557
Lin - 150	0.476	0.0653	0.2877	0.0596	0.4098	0.0669	0.3782	0.0722	0.462	0.0542	0.4151	0.066	0.4446	0.0339
Baseline - 50	0.5657	0.0815	0.3413	0.0775	0.4807	0.0943	0.471	0.099	0.5211	0.0806	0.4938	0.0917	0.472	0.0506
Baseline - 100	0.5128	0.0773	0.3005	0.0904	0.4385	0.0789	0.4154	0.0893	0.4832	0.0641	0.4456	0.0778	0.4528	0.0547
Baseline - 150	0.4713	0.0877	0.2463	0.1066	0.3849	0.1051	0.3548	0.1121	0.4369	0.0907	0.3902	0.1038	0.4206	0.0634
Baseline_LCA – 50	0.7597	0.0339	0.5016	0.0631	0.5933	0.0371	0.5925	0.0384	0.6147	0.0346	0.6033	0.0361	0.5907	0.0484
Baseline_LCA - 100	0.7272	0.058	0.4855	0.0777	0.5782	0.0443	0.5691	0.0491	0.5986	0.0386	0.5833	0.0437	0.5809	0.0575
Baseline_LCA - 150	0.6959	0.0649	0.4547	0.0807	0.5348	0.0524	0.5248	0.0579	0.5534	0.0453	0.5386	0.0519	0.5558	0.058
Baseline+Desc - 50 (0.7,0.3)	0.5693	0.0376	0.3583	0.0541	0.5	0.055	0.4942	0.0502	0.5325	0.0601	0.5124	0.0535	0.4775	0.0454
Baseline+Desc - 100 (0.7,0.3)	0.5317	0.092	0.3217	0.1064	0.4629	0.0991	0.444	0.1087	0.5024	0.078	0.4697	0.0975	0.4659	0.0606
Baseline+Desc - 150 (0.7,0.3)	0.552	0.0714	0.3393	0.0879	0.4904	0.0774	0.4658	0.0869	0.5294	0.0621	0.4947	0.0765	0.4812	0.0519
GOntoSim - 50(0.7,0.3)	0.708	0.0767	0.4355	0.081	0.5629	0.0728	0.5569	0.0744	0.5922	0.067	0.5738	0.0707	0.5425	0.0595
GOntoSim - 100 (0.7,0.3)	0.7278	0.0575	0.4849	0.0822	0.5769	0.0551	0.5682	0.0602	0.597	0.0484	0.582	0.0544	0.5801	0.0609
GOntoSim - 150(0.7,0.3)	0.7323	0.0648	0.5008	0.0816	0.5858	0.0635	0.576	0.0665	0.6032	0.0598	0.5891	0.063	0.5924	0.0634
Baseline+Desc – 50 (0.5,0.5)	0.5533	0.0875	0.3602	0.0998	0.4934	0.0987	0.481	0.0978	0.5359	0.0925	0.5066	0.0955	0.4893	0.0731
Baseline+Desc - 100 (0.5,0.5)	0.5752	0.0659	0.3843	0.07	0.5259	0.0621	0.5031	0.0658	0.5651	0.06	0.5318	0.0612	0.5106	0.0511
Baseline+Desc - 150 (0.5,0.5)	0.5633	0.0983	0.3697	0.1181	0.5031	0.0974	0.4769	0.1126	0.5451	0.0754	0.5073	0.0964	0.5066	0.0748
GOntoSim - 50 (0.5,0.5)	0.7183	0.0776	0.475	0.1071	0.5785	0.0717	0.5768	0.0744	0.6019	0.0651	0.589	0.0697	0.5705	0.0833
GOntoSim - 100 (0.5,0.5)	0.7398	0.0874	0.5034	0.1175	0.5872	0.0718	0.5802	0.0788	0.6053	0.0626	0.5922	0.0708	0.596	0.0864
GOntoSim - 150 (0.5,0.5)	0.7527	0.0761	0.5248	0.0985	0.6023	0.0731	0.5944	0.0769	0.6171	0.0677	0.6055	0.0725	0.6108	0.0769

Table 2. NONIEA annotations for EC Level 1 - Biological Process

BP (Level 1)	purity_score		ARI_Score		AMI_Score		homogeneity_score		completeness_score		v_measure_score		fowlkes_mallows_score	
samples * 10	Mean	Std-dev	Mean	Std-dev	Mean	Std-dev	Mean	Std-dev	Mean	Std-dev	Mean	Std-dev	Mean	Std-dev
Wang - 50	0.281	0.0296	0.027	0.0176	0.0685	0.0332	0.0773	0.0264	0.1335	0.0359	0.0976	0.0306	0.3042	0.0133
Wang - 100	0.2682	0.0346	0.0292	0.0202	0.067	0.0282	0.0661	0.0254	0.1092	0.0257	0.0817	0.0266	0.3032	0.0138
Wang - 150	0.2599	0.0209	0.0249	0.0117	0.0616	0.0184	0.0571	0.0153	0.0959	0.0215	0.0715	0.0179	0.3034	0.0086
Baseline - 50	0.2547	0.0151	0.0126	0.0071	0.0551	0.0148	0.0649	0.0117	0.1406	0.0184	0.0884	0.0136	0.3264	0.0124
Baseline - 100	0.2488	0.0158	0.0126	0.0048	0.0518	0.0165	0.0513	0.0135	0.0998	0.0188	0.0676	0.0159	0.3151	0.0095
Baseline - 150	0.2441	0.0174	0.0103	0.0045	0.0448	0.012	0.0417	0.0104	0.0826	0.013	0.0552	0.0115	0.3184	0.0133
Baseline_LCA – 50	0.322	0.0331	0.059	0.0295	0.0946	0.0331	0.1066	0.0315	0.1423	0.0319	0.1211	0.0313	0.2805	0.0219
Baseline_LCA - 100	0.2827	0.0215	0.0396	0.0153	0.0644	0.0191	0.0665	0.0171	0.0959	0.0199	0.0782	0.0183	0.2766	0.0197
Baseline_LCA - 150	0.2894	0.0105	0.0434	0.0136	0.0766	0.0135	0.0718	0.0118	0.1068	0.0163	0.0857	0.0132	0.2865	0.0171
Baseline+Desc - 50	0.2623	0.023	0.0128	0.0077	0.0559	0.0251	0.0659	0.0184	0.1364	0.0376	0.0886	0.024	0.322	0.0098
Baseline+Desc - 100	0.2512	0.0229	0.0119	0.0071	0.055	0.0231	0.0532	0.019	0.1061	0.0262	0.0707	0.0222	0.3212	0.0081
Baseline+Desc - 150	0.2497	0.0143	0.0115	0.0028	0.0558	0.0119	0.0495	0.0091	0.1006	0.0177	0.0662	0.0117	0.323	0.0087
GOntoSim - 50	0.2977	0.0235	0.0437	0.0152	0.0781	0.0215	0.0912	0.0194	0.1253	0.0202	0.1052	0.0199	0.2762	0.0175
GOntoSim - 100	0.3045	0.0237	0.0503	0.0212	0.0846	0.0205	0.0858	0.0195	0.1136	0.019	0.0975	0.0197	0.275	0.0136
GOntoSim - 150	0.2897	0.0188	0.0396	0.0137	0.0693	0.0157	0.0669	0.0133	0.0947	0.0191	0.0783	0.0156	0.272	0.0165

Table 3. IEA annotations for EC Level 1 - Molecular Function

MF(Level 1) - IEA	Purity Score		ARI Score		AMI Score		Homogeneity Score		Completeness Score		V-Measure Score		Fowlkes Mallows Score	
samples * 10	Mean	Std-dev	Mean	Std-dev	Mean	Std-dev	Mean	Std-dev	Mean	Std-dev	Mean	Std-dev	Mean	Std-dev
Wang - 50	0.6447	0.0723	0.4268	0.0797	0.5602	0.0659	0.5392	0.0776	0.6107	0.0562	0.5712	0.0641	0.5438	0.0531
Wang - 100	0.6357	0.0864	0.4536	0.126	0.5733	0.1099	0.5397	0.1127	0.6263	0.1039	0.5786	0.1083	0.5702	0.0899
Wang - 150	0.602	0.0735	0.4026	0.0925	0.5292	0.0926	0.4936	0.095	0.5831	0.096	0.5332	0.0917	0.5302	0.0688
Wang - 250	0.5731	0.0844	0.4023	0.0792	0.5475	0.0904	0.4844	0.0928	0.6423	0.0968	0.5498	0.0899	0.549	0.0551
Wang - 400	0.4764	0.1176	0.246	0.1338	0.3896	0.1381	0.3336	0.1475	0.4979	0.1048	0.3918	0.1374	0.454	0.0707
Wang - 500	0.4805	0.1236	0.2544	0.1436	0.3911	0.153	0.3283	0.1466	0.5111	0.1438	0.3929	0.1524	0.4668	0.0778
GOGO - 50	0.708	0.0587	0.5216	0.09	0.6363	0.0714	0.6312	0.0755	0.6606	0.0636	0.6454	0.0695	0.6086	0.0699
GOGO - 100	0.6908	0.0577	0.5129	0.0788	0.6213	0.0642	0.5999	0.0671	0.6552	0.0617	0.626	0.0634	0.6061	0.0614
GOGO - 150	0.5378	0.1022	0.3237	0.1124	0.4634	0.1256	0.4211	0.1267	0.533	0.1132	0.4682	0.1241	0.4835	0.0732
GOGO - 250	0.5402	0.0822	0.3195	0.105	0.4586	0.1071	0.414	0.1071	0.5248	0.1082	0.4615	0.1064	0.4787	0.071
GOGO - 400	0.4602	0.0629	0.2515	0.087	0.3826	0.0848	0.3198	0.086	0.4887	0.0789	0.3847	0.0845	0.4504	0.0554
GOGO - 500	0.4873	0.0759	0.2587	0.0853	0.3987	0.1125	0.3394	0.1038	0.4933	0.122	0.4003	0.1121	0.4517	0.0563
Resnik - 500	0.7039	0.0743	0.621	0.1018	0.7782	0.0522	0.6993	0.0747	0.8827	0.0252	0.7788	0.052	0.7244	0.0648
Lin - 500	0.8197	0.0074	0.7863	0.0176	0.8797	0.0171	0.8281	0.0166	0.939	0.0182	0.88	0.0171	0.835	0.014
Baseline - 50	0.6957	0.0706	0.5146	0.0974	0.6358	0.068	0.6205	0.0802	0.6741	0.0543	0.6449	0.066	0.6088	0.0678
Baseline - 100	0.669	0.0559	0.4799	0.0738	0.6196	0.0836	0.5812	0.0693	0.676	0.1048	0.6244	0.0826	0.5899	0.0645
Baseline - 150	0.6241	0.0521	0.4261	0.0788	0.5897	0.0706	0.5359	0.0716	0.6662	0.0715	0.5932	0.0699	0.5587	0.0609
Baseline - 250	0.6507	0.0884	0.4654	0.1414	0.6029	0.1226	0.5522	0.1257	0.6706	0.1142	0.6049	0.122	0.5874	0.1053
Baseline - 400	0.6324	0.0801	0.4452	0.0994	0.5822	0.0891	0.5322	0.0993	0.6491	0.0698	0.5835	0.0888	0.5715	0.0669
Baseline - 500	0.6512	0.0514	0.4833	0.0656	0.6116	0.0549	0.5618	0.0586	0.6756	0.0591	0.6126	0.0548	0.5971	0.0485
Baseline_LCA - 50	0.8397	0.0763	0.6977	0.1099	0.785	0.0467	0.7647	0.0662	0.8201	0.0315	0.7903	0.0455	0.7599	0.0792
Baseline_LCA - 100	0.8103	0.033	0.6406	0.0591	0.7446	0.0373	0.7228	0.0339	0.7753	0.0481	0.7477	0.0369	0.7105	0.0476
Baseline_LCA - 150	0.792	0.078	0.6515	0.1345	0.7678	0.0734	0.7311	0.0878	0.8147	0.0573	0.7698	0.0727	0.729	0.0971
Baseline_LCA - 250	0.8187	0.0671	0.7105	0.0982	0.7954	0.0506	0.7594	0.0681	0.8386	0.0319	0.7964	0.0504	0.7729	0.0729
Baseline_LCA - 400	0.8753	0.0796	0.7687	0.1265	0.8177	0.063	0.8011	0.0777	0.8375	0.0508	0.8183	0.0628	0.8141	0.0979
Baseline_LCA - 500	0.8223	0.0758	0.6911	0.1346	0.7776	0.0764	0.7465	0.0894	0.8142	0.0659	0.7781	0.0762	0.7558	0.1021
Desc–50(0.7,0.3)	0.718	0.0607	0.5302	0.0772	0.6566	0.0612	0.6406	0.0669	0.6929	0.0532	0.6653	0.0594	0.6216	0.0576
Baseline+Desc - 100(0.7,0.3)	0.6813	0.0485	0.5037	0.077	0.6357	0.0677	0.593	0.0699	0.6966	0.0623	0.6403	0.0667	0.611	0.0566
Baseline+Desc - 150(0.7,0.3)	0.7228	0.1034	0.5651	0.1486	0.6781	0.1115	0.64	0.128	0.7317	0.0869	0.6808	0.1105	0.6605	0.1031
Baseline+Desc - 250(0.7,0.3)	0.7157	0.0733	0.5864	0.0896	0.7079	0.0684	0.6537	0.0792	0.7779	0.0569	0.7094	0.068	0.6793	0.0633
Baseline+Desc - 400(0.7,0.3)	0.678	0.1062	0.547	0.1387	0.682	0.1207	0.6163	0.1289	0.7734	0.1053	0.6831	0.1203	0.6603	0.0906
Baseline+Desc - 500(0.7,0.3)	0.6691	0.0812	0.5269	0.1232	0.6552	0.0977	0.5966	0.1067	0.7322	0.0854	0.6561	0.0974	0.6372	0.0888
GOntoSim - 50(0.7,0.3)	0.8063	0.051	0.6498	0.0587	0.7564	0.0386	0.7339	0.0446	0.7946	0.0402	0.7625	0.0376	0.7194	0.0437
GOntoSim - 100(0.7,0.3)	0.8002	0.0698	0.6288	0.1155	0.7516	0.0579	0.7233	0.0712	0.7906	0.0452	0.7548	0.0571	0.707	0.0853
GOntoSim - 150(0.7,0.3)	0.8041	0.0812	0.657	0.1321	0.7699	0.07	0.7385	0.0867	0.8099	0.0512	0.7718	0.0694	0.7311	0.098
GOntoSim - 250(0.7,0.3)	0.806	0.073	0.6603	0.1208	0.7697	0.0637	0.7391	0.0775	0.8067	0.0499	0.7709	0.0634	0.7323	0.0909
GOntoSim - 400(0.7,0.3)	0.8898	0.0883	0.7975	0.1355	0.8533	0.0724	0.8359	0.0939	0.8743	0.0496	0.8537	0.0722	0.8387	0.1032
GOntoSim - 500(0.7,0.3)	0.8229	0.0654	0.703	0.0914	0.8022	0.0455	0.7704	0.0593	0.8393	0.0376	0.8027	0.0454	0.7645	0.0693
Baseline+Desc – 50(0.5,0.5)	0.7927	0.1196	0.6347	0.17	0.7322	0.1323	0.7215	0.1389	0.7581	0.1172	0.7389	0.1288	0.7041	0.1328
Baseline+Desc - 100(0.5,0.5)	0.7293	0.0823	0.5659	0.1051	0.6772	0.0719	0.6498	0.0833	0.7178	0.06	0.6812	0.0709	0.655	0.0766
Baseline+Desc - 150(0.5,0.5)	0.6856	0.0961	0.5184	0.1229	0.6529	0.0949	0.6077	0.1079	0.7178	0.082	0.6559	0.0939	0.6288	0.0804
Baseline+Desc - 250(0.5,0.5)	0.7	0.0764	0.5489	0.1137	0.685	0.0793	0.6352	0.0883	0.7491	0.0648	0.6866	0.0788	0.6506	0.08
Baseline+Desc - 400(0.5,0.5)	0.6765	0.0927	0.5566	0.1224	0.6988	0.0988	0.6318	0.1134	0.7908	0.0831	0.6998	0.0984	0.667	0.0841
Baseline+Desc - 500(0.5,0.5)	0.7135	0.1094	0.6119	0.1445	0.7262	0.1171	0.6693	0.1314	0.802	0.0878	0.7269	0.1168	0.7053	0.0937
GOntoSim - 50(0.5,0.5)	0.8537	0.0439	0.6961	0.0861	0.7892	0.0599	0.778	0.0611	0.8122	0.0604	0.7944	0.0584	0.7523	0.069
GOntoSim - 100(0.5,0.5)	0.7763	0.0682	0.5941	0.1328	0.7316	0.0906	0.6982	0.0953	0.777	0.0856	0.735	0.0894	0.6816	0.1003
GOntoSim - 150(0.5,0.5)	0.8007	0.0575	0.6645	0.096	0.7829	0.0577	0.7472	0.0659	0.8272	0.0507	0.7848	0.0572	0.7369	0.071
GOntoSim - 250(0.5,0.5)	0.8416	0.0938	0.72	0.1529	0.8129	0.0823	0.7847	0.1024	0.8475	0.0613	0.8138	0.0819	0.7801	0.1156
GOntoSim - 400(0.5,0.5)	0.839	0.0736	0.7446	0.1045	0.8298	0.058	0.7974	0.0741	0.8676	0.0442	0.8303	0.0578	0.7986	0.0796
GOntoSim - 500(0.5,0.5)	0.9424	0.0671	0.9052	0.0855	0.9266	0.0478	0.9154	0.0648	0.9398	0.0348	0.9268	0.0477	0.924	0.0658

Table 4. NONIEA annotations for EC Level 1 - Molecular Function - **with Tanimoto Coefficient**

MF(Level 1)	purity_score		ARI_Score		AMI_Score		homogeneity_score		completeness_score		v_measure_score		fowlkes_mallows_score	
**samples * 10**	**Mean**	**Std-dev**	**Mean**	**Std-dev**	**Mean**	**Std-dev**	**Mean**	**Std-dev**	**Mean**	**Std-dev**	**Mean**	**Std-dev**	**Mean**	**Std-dev**
**Baseline - 50**	0.5657	0.0815	0.3413	0.0775	0.4807	0.0943	0.471	0.099	0.5211	0.0806	0.4938	0.0917	0.472	0.0506
**Baseline - 100**	0.5128	0.0773	0.3005	0.0904	0.4385	0.0789	0.4154	0.0893	0.4832	0.0641	0.4456	0.0778	0.4528	0.0547
**Baseline - 150**	0.4713	0.0877	0.2463	0.1066	0.3849	0.1051	0.3548	0.1121	0.4369	0.0907	0.3902	0.1038	0.4206	0.0634
**Baseline_TC - 150**	0.4986	0.0896	0.284	0.1047	0.4189	0.0894	0.3874	0.1019	0.4734	0.0684	0.4239	0.0884	0.4502	0.0607
**Baseline_LCA - 50**	0.7597	0.0339	0.5016	0.0631	0.5933	0.0371	0.5925	0.0384	0.6147	0.0346	0.6033	0.0361	0.5907	0.0484
**Baseline_LCA - 100**	0.7272	0.058	0.4855	0.0777	0.5782	0.0443	0.5691	0.0491	0.5986	0.0386	0.5833	0.0437	0.5809	0.0575
**Baseline_LCA - 150**	0.6959	0.0649	0.4547	0.0807	0.5348	0.0524	0.5248	0.0579	0.5534	0.0453	0.5386	0.0519	0.5558	0.058
**Baseline+Desc – 50 (0.7,0.3)**	0.5693	0.0376	0.3583	0.0541	0.5	0.055	0.4942	0.0502	0.5325	0.0601	0.5124	0.0535	0.4775	0.0454
**Baseline+Desc - 100 (0.7,0.3)**	0.5317	0.092	0.3217	0.1064	0.4629	0.0991	0.444	0.1087	0.5024	0.078	0.4697	0.0975	0.4659	0.0606
**Baseline+Desc - 150 (0.7,0.3)**	0.552	0.0714	0.3393	0.0879	0.4904	0.0774	0.4658	0.0869	0.5294	0.0621	0.4947	0.0765	0.4812	0.0519
**Baseline+Desc_TC - 150 (0.7,0.3)**	0.547	0.0443	0.3477	0.0546	0.4848	0.0535	0.4594	0.0582	0.5238	0.0473	0.4891	0.0529	0.4854	0.0332
**GOntoSim - 50 (0.7,0.3)**	0.708	0.0767	0.4355	0.081	0.5629	0.0728	0.5569	0.0744	0.5922	0.067	0.5738	0.0707	0.5425	0.0595
**GOntoSim - 100 (0.7,0.3)**	0.7278	0.0575	0.4849	0.0822	0.5769	0.0551	0.5682	0.0602	0.597	0.0484	0.582	0.0544	0.5801	0.0609
**GOntoSim - 150 (0.7,0.3)**	0.7323	0.0648	0.5008	0.0816	0.5858	0.0635	0.576	0.0665	0.6032	0.0598	0.5891	0.063	0.5924	0.0634
**GOntoSim_TC - 150 (0.7,0.3)**	0.7056	0.0816	0.4718	0.0998	0.5737	0.0669	0.5592	0.0736	0.5967	0.058	0.5771	0.0662	0.5733	0.0738
**Baseline+Desc – 50 (0.5,0.5)**	0.5533	0.0875	0.3602	0.0998	0.4934	0.0987	0.481	0.0978	0.5359	0.0925	0.5066	0.0955	0.4893	0.0731
**Baseline+Desc - 100** (0.5,0.5)	0.5752	0.0659	0.3843	0.07	0.5259	0.0621	0.5031	0.0658	0.5651	0.06	0.5318	0.0612	0.5106	0.0511
**Baseline+Desc - 150** (0.5,0.5)	0.5633	0.0983	0.3697	0.1181	0.5031	0.0974	0.4769	0.1126	0.5451	0.0754	0.5073	0.0964	0.5066	0.0748
**Baseline+Desc_TC - 150 (0.5,0.5)**	0.5439	0.0493	0.3345	0.0688	0.4679	0.0571	0.4417	0.0638	0.51	0.0534	0.4724	0.0566	0.4776	0.0434
**GOntoSim - 50 (0.5,0.5)**	0.7183	0.0776	0.475	0.1071	0.5785	0.0717	0.5768	0.0744	0.6019	0.0651	0.589	0.0697	0.5705	0.0833
**GOntoSim - 100 (0.5,0.5)**	0.7398	0.0874	0.5034	0.1175	0.5872	0.0718	0.5802	0.0788	0.6053	0.0626	0.5922	0.0708	0.596	0.0864
**GOntoSim - 150 (0.5,0.5)**	0.7527	0.0761	0.5248	0.0985	0.6023	0.0731	0.5944	0.0769	0.6171	0.0677	0.6055	0.0725	0.6108	0.0769
**GOntoSim_TC - 150 (0.5,0.5)**	0.7438	0.0966	0.518	0.1412	0.6022	0.0903	0.5919	0.0977	0.6201	0.0803	0.6054	0.0895	0.609	0.1065

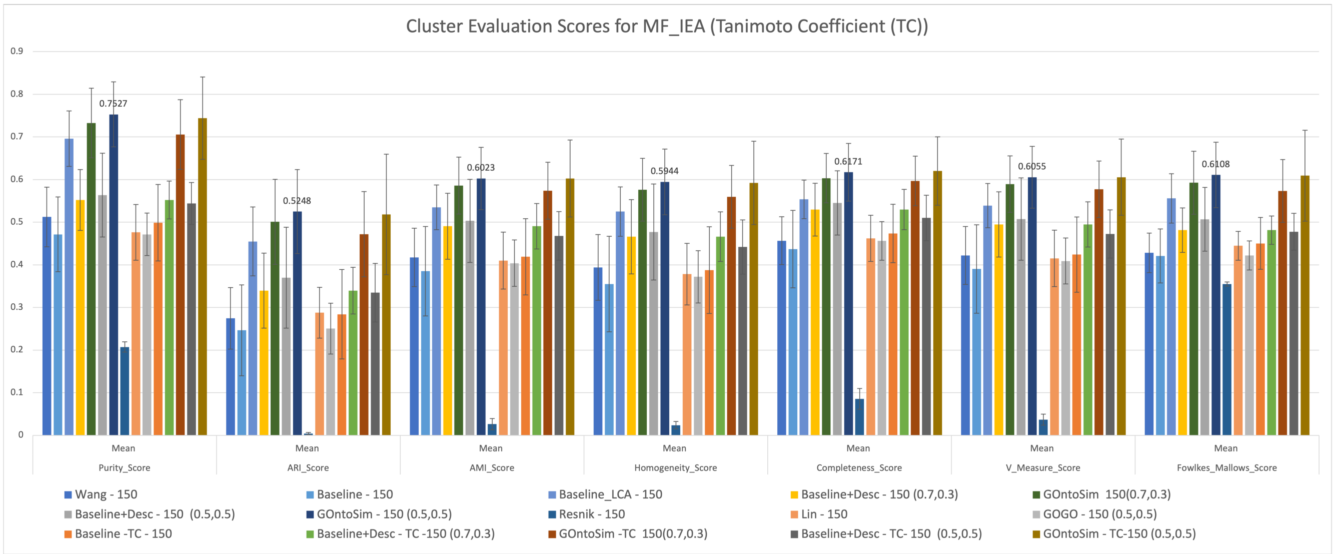

Figure 1. Cluster Evaluation at EC Level 1 for MF annotations (NONIEA). Baseline, Baseline+Desc, and GOntoSim Methods implemented with Tanimoto Coefficient to check for optimizations.

Table 5. IEA annotations for EC Level 1 - Molecular Function - **with Tanimoto Coefficient**

MF(Level 1)	purity_score		ARI_Score		AMI_Score		homogeneity_score		completeness_score		v_measure_score		fowlkes_mallows_score	
**samples * 10**	**Mean**	**Std-dev**	**Mean**	**Std-dev**	**Mean**	**Std-dev**	**Mean**	**Std-dev**	**Mean**	**Std-dev**	**Mean**	**Std-dev**	**Mean**	**Std-dev**
**Wang - 500**	0.4805	0.1236	0.2544	0.1436	0.3911	0.153	0.3283	0.1466	0.5111	0.1438	0.3929	0.1524	0.4668	0.0778
**GOGO - 500**	0.4873	0.0759	0.2587	0.0853	0.3987	0.1125	0.3394	0.1038	0.4933	0.122	0.4003	0.1121	0.4517	0.0563
**Resnik - 500**	0.7039	0.0743	0.621	0.1018	0.7782	0.0522	0.6993	0.0747	0.8827	0.0252	0.7788	0.052	0.7244	0.0648
**Lin - 500**	0.8197	0.0074	0.7863	0.0176	0.8797	0.0171	0.8281	0.0166	0.939	0.0182	0.88	0.0171	0.835	0.014
**Baseline - 500**	0.6512	0.0514	0.4833	0.0656	0.6116	0.0549	0.5618	0.0586	0.6756	0.0591	0.6126	0.0548	0.5971	0.0485
**Baseline_TC - 500**	0.4763	0.1116	0.2753	0.1088	0.3909	0.1075	0.3405	0.1145	0.4699	0.0902	0.3926	0.107	0.4594	0.0644
**GOntoSim - 500(0.7,0.3)**	0.8229	0.0654	0.703	0.0914	0.8022	0.0455	0.7704	0.0593	0.8393	0.0376	0.8027	0.0454	0.7645	0.0693
**GOntoSim_TC - 500**	0.9221	0.0666	0.839	0.12	0.8726	0.0705	0.8655	0.0815	0.881	0.0593	0.8729	0.0704	0.8689	0.0944
**GOntoSim - 500(0.5,0.5)**	0.9424	0.0671	0.9052	0.0855	0.9266	0.0478	0.9154	0.0648	0.9398	0.0348	0.9268	0.0477	0.924	0.0658
**GOntoSim_TC - 500 (0.5,0.5)**	0.8531	0.0568	0.7836	0.0677	0.8653	0.034	0.8335	0.0467	0.9014	0.0307	0.8656	0.034	0.8291	0.0507
**Baseline_LCA - 500**	0.8223	0.0758	0.6911	0.1346	0.7776	0.0764	0.7465	0.0894	0.8142	0.0659	0.7781	0.0762	0.7558	0.1021
**Baseline+Desc - 500(0.7,0.3)**	0.6691	0.0812	0.5269	0.1232	0.6552	0.0977	0.5966	0.1067	0.7322	0.0854	0.6561	0.0974	0.6372	0.0888
**Baseline+Desc_TC - 500(0.7,0.3)**	0.8229	0.0654	0.703	0.0914	0.8022	0.0455	0.7704	0.0593	0.8393	0.0376	0.8027	0.0454	0.7645	0.0693
**Baseline+Desc - 500(0.5,0.5)**	0.7135	0.1094	0.6119	0.1445	0.7262	0.1171	0.6693	0.1314	0.802	0.0878	0.7269	0.1168	0.7053	0.0937
**Baseline+Desc_TC - 500 (0.5,0.5)**	0.5461	0.1032	0.3233	0.1109	0.453	0.1178	0.4085	0.1205	0.5168	0.1057	0.4544	0.1174	0.4852	0.065

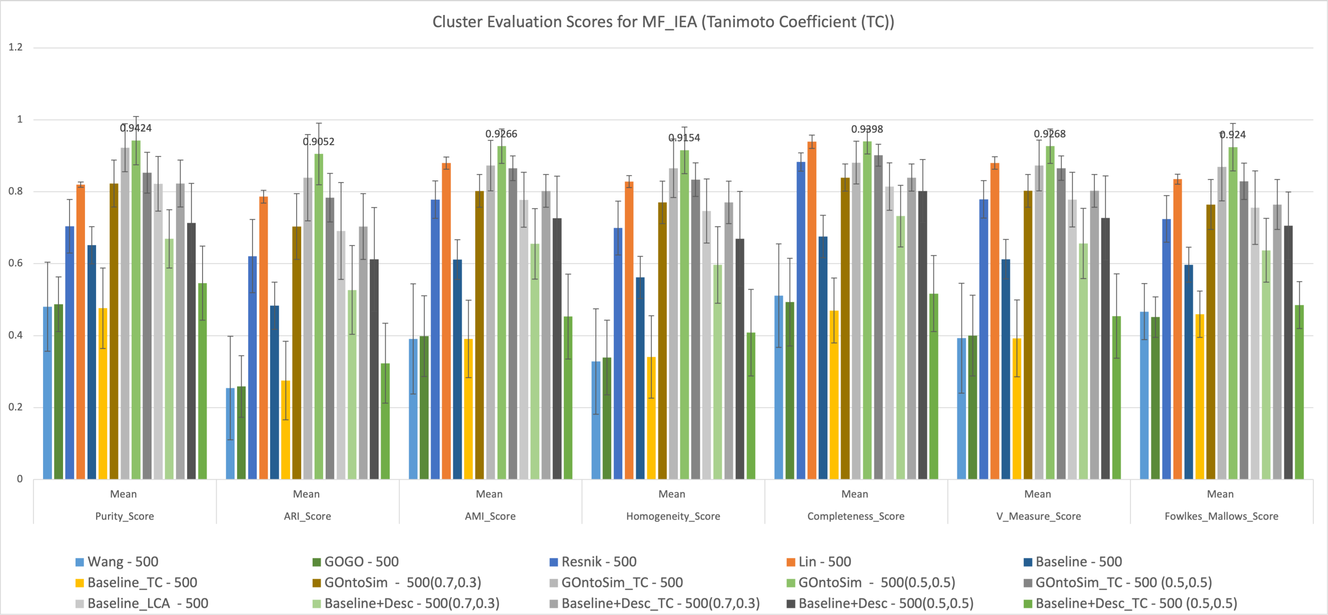

Figure 2. Cluster Evaluation at EC Level 1 for MF annotations (IEA). Baseline, Baseline+Desc, and GOntoSim Methods implemented with Tanimoto Coefficient to check for optimizations.
